# Application of Double‐Colored Die‐Stone as an Alternative Approach for Achieving Superior Finish Line Precision in Dental Casts: A Comparative In Vitro Study

**DOI:** 10.1155/ijod/8947455

**Published:** 2026-01-16

**Authors:** Ahmad Toumaj, Akram Ghannadpour, Mahtab Mottaghi, Mirkamal Hosseiny, Seyed Mehdi Ziaei, Mona Saligherad, Kiarash Kiani

**Affiliations:** ^1^ Department of Prosthodontics, Faculty of Dentistry, Islamic Azad University of Medical Sciences, Tehran, Iran, iautmu.ac.ir; ^2^ School of Dentistry, Tehran University of Medical Sciences, Tehran, Iran, tums.ac.ir; ^3^ School of Dentistry, Mashhad University of Medical Sciences, Mashhad, Iran, mums.ac.ir; ^4^ School of Dentistry, Hamadan University of Medical Sciences, Hamadan, Iran, umsha.ac.ir; ^5^ School of Dentistry, Tehran Medical Sciences, Islamic Azad University, Tehran, Iran, iautmu.ac.ir

**Keywords:** chamfer, conventional technique, digital technique, finish line

## Abstract

**Introduction:**

Determining the finish line on the master cast is essential to create proper restoration. We aimed to compare the finish line accuracy in casts created using the conventional technique, digital technique, and an alternative approach called the double‐colored die‐stone (DCDS) technique.

**Methods:**

The tooth 24 in an upper jaw typodont was prepared with a chamfer finish line. Twenty impressions were taken from the typodont and were divided into conventional (*n* = 10) and DCDS (*n* = 10). Ten digital impressions were taken using an intraoral scanner, and a 3D printer made the digital cast. We applied a single‐color die‐stone in the conventional method, while we used two colors of die‐stone in the DCDS group. We assessed the finish lines, focusing on the area of valid distance, distance standard deviation, integrated absolute distance, and mean distance. A reference model was created to evaluate the area of valid distance across different techniques. Statistical analysis was performed using the Kruskal–Wallis and Wilcoxon rank‐sum tests.

**Results:**

The area of valid distance was significantly higher in the DCDS group than in the digital group (*p*  < 0.001). The integrated absolute distance was notably greater in the digital group than in the conventional group (*p*  < 0.001). We revealed significant differences in the area of valid distance between the digital, conventional, and DCDS groups compared to the reference model (*p*  = 0.002, *p*  = 0.002, and *p*  = 0.011, respectively). However, the DCDS group demonstrated the closest alignment with the reference model.

**Conclusions:**

This study suggests that the DCDS technique could offer an effective alternative to the conventional method.

**Clinical Significance:**

The current study highlights the DCDS as a reliable alternative to conventional and digital methods, exhibiting acceptable precision in replicating the finish line. This technology enhances the visualization of the finish line, improving prosthetics’ fabrication process.

**Summary**

• We present a modified casting method employing double‐colored die‐stone (DCDS), which leads to superior results compared to conventional and digital techniques.

• Employing this method demonstrated our commitment to minimizing errors in determining the finishing line of the die before and after ditching, thereby enhancing the precision of dental technicians’ work.

## 1. Introduction

Accurate identification of the finish line is a key step in creating a well‐prepared restoration [1]. The finish line marks the boundary between prepared and unprepared regions in a tooth that defines the margin of the restoration. It should form a smooth and continuous transition from one surface to another [2]. The finish line of the master cast should be easily visible to help technicians precisely determine the correct cervical margin of the prosthesis [3].

Currently, two options are available to create impressions and casts of dental arches: the digital technique and the conventional technique [4]. The conventional technique offers various benefits, making it a valuable option in dental treatment. This approach ensures sufficient stability and accuracy, enabling precise marginal fit and serving as a legal document. Another notable advantage is that specialized equipment is unnecessary [5, 6]. Despite these advantages, a ditching procedure in the conventional approach, which identifies the margin of the restoration, could be prone to error [7].

The digital technique has evolved with intraoral scanners and 3D printers [6]. The potential benefits of digital impressions and workflows are eliminating manufacturing stages that could lead to misfits, decreasing the transfer between clinical and dental laboratories, and improving the patient’s comfort [8]. A further benefit is the ability to digitally ditch the die with margin‐defining software, automatically allowing the scanning program to recognize the prepared finish line [9, 10]. Although the digital technique could enhance the workflow, several limitations affect the application of the digital method. Initially, digital systems require substantial technical proficiency and expertise. Unlike the conventional method, which tends to be simple, the digital approach demands users to learn special technology and software [11, 12]. Specialized digital equipment could be costly, restricting accessibility for some dental facilities. There is always a discrepancy between technological advancements and clinical integration, partly due to the cost [12]. Additionally, a subgingival finishing line can result in poor scan accuracy due to blood, saliva, and gingival obstructions [13].

To address these challenges, we introduced the double‐colored die‐stone (DCDS) technique, which modifies the conventional technique. This approach uses two different die‐stone colors to improve the finish line identification throughout the casting process. By using the core principles of conventional casting, the DCDS technique aims to enhance the finish line detection without requiring digital equipment. Therefore, this technique could be affordable in dental laboratories with limited access to advanced digital equipment.

Various studies have compared the accuracy of digital and conventional methods, and they regularly show better results with the digital method [5, 6, 8]. This study aims to assess the accuracy of the DCDS technique compared to conventional and digital methods. We evaluate how closely each technique replicates a reference model.

## 2. Materials and Methods

This in vitro pilot study examines three groups of dies fabricated using different casting techniques. Thirty impressions were taken from the prepared maxillary arch (*n* = 10), providing enough information to determine effect size and guide future power calculation.

### 2.1. Tooth Preparation

An upper jaw typodont model (Basic Study Model, Kavo Dental GmbH) was used, with the left first premolar prepared with a 1 mm‐wide continuous chamfer and a 90° cavosurface angle. The preparation process included a total occlusal convergence of 20° and a 1.5 mm occlusal reduction at the center of the occlusal surface. A high‐speed handpiece (KaVo Bella Torque Mini; KaVo) with diamond burs (NTI Diamond Instruments) was used to create a proper finish line at the gingival level. Figure 1A shows an upper jaw typodont with a prepared left first premolar tooth.

Figure 1(A) Maxillary typodont with prepared left first premolar. (B) Putty‐wash impression of prepared maxillary typodont. (C) Application of first layer of die‐stone in DCDS technique. (D) Application of second layer of die‐stone in DCDS technique.

**Figure:**
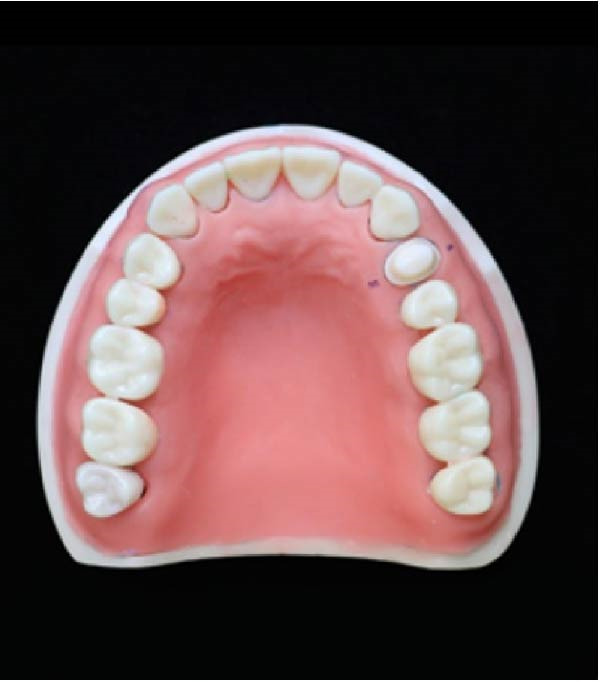
(A)

**Figure:**
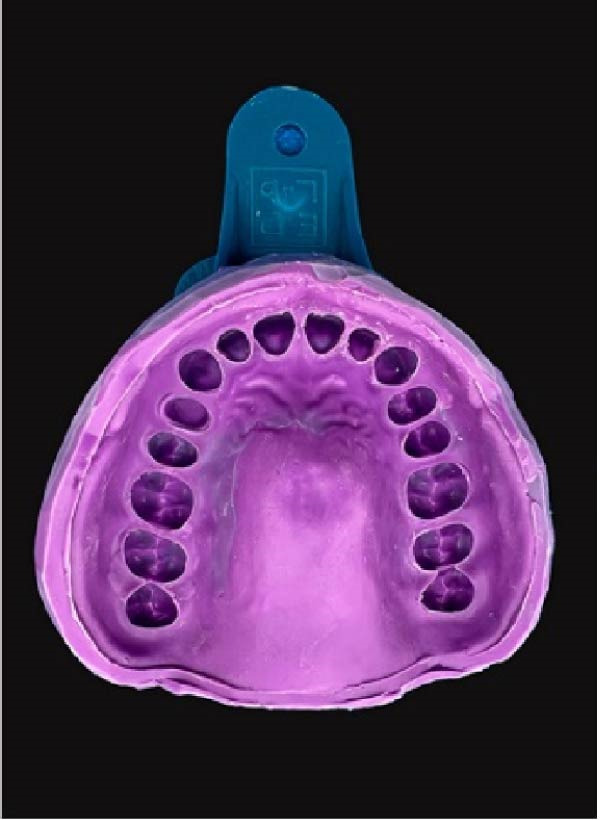
(B)

**Figure:**
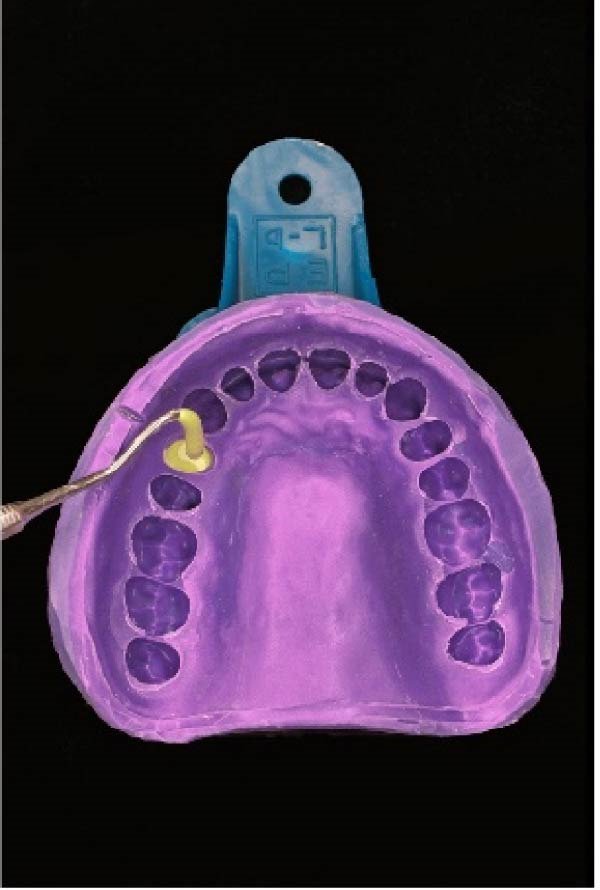
(C)

**Figure:**
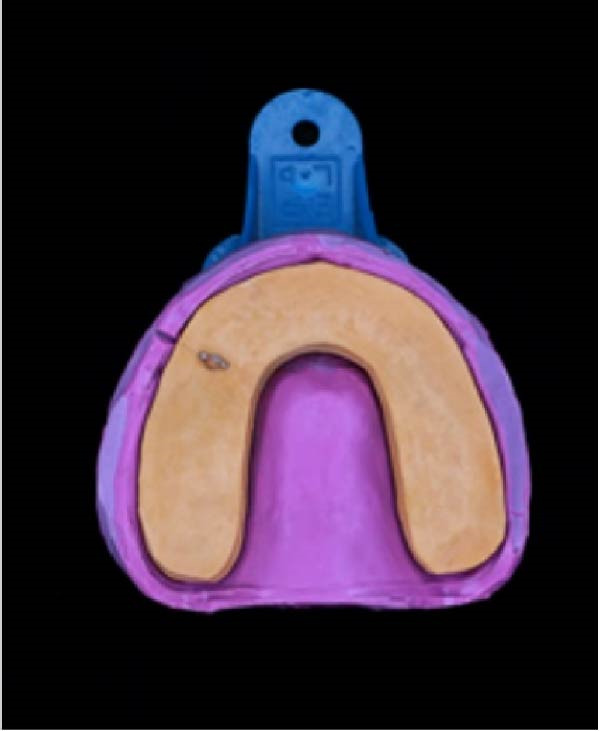
(D)

### 2.2. DCDS Technique

Ten impressions were taken using a two‐step putty‐wash technique. Once the putty had been set, the excess material was trimmed, and 2 mm sluiceways were carved to create space for wash material. Following the wash application, the trays were kept for 12 min with an extended setting time to adjust room temperature. Figure 1B illustrates a recently obtained impression. Following the formation of the impression, they were inspected for any defects. The buccal and lingual surfaces of the prepared teeth were marked transversely, and the impressions were covered with three layers of surfactant.

The impressions were poured with Type IV die‐stone. The materials were gradually poured into the prepared regions using a vibrator to ensure smooth flow and eliminate air bubbles.

#### 2.2.1. The First Layer of Die‐Stone

A small amount of colored die‐stone was poured into the impression. The replica of the prepared tooth was completely covered up to the finish line. The backside of a shepherd’s hook end of a dental explorer was used to remove the excess material over the finish line. The die‐stone was allowed to be set until the surface became matte (Figure 1C).

#### 2.2.2. The Second Layer of Die‐Stone

The second layer of die‐stone was poured up to the impression border, ensuring the visibility of the transverse lingual and buccal lines. A brass pin was positioned parallel to these surfaces and allowed to set for 30–45 min. Afterward, we coated the die‐stone surface with a separating agent (Figure 1D).

#### 2.2.3. Base Pouring

A base layer of plaster (Moldano Dental Stone, Bayer) was added to complete the cast, and a plaster saw was used to separate the dies (Figure 2A).

Figure 2(A) Dental cast with a die prepared using DCDS technique. (B) Die prepared using DCDS technique. (C) Dental cast with die prepared using conventional technique. (D) Resin cast printed by the 3D printer.

**Figure:**
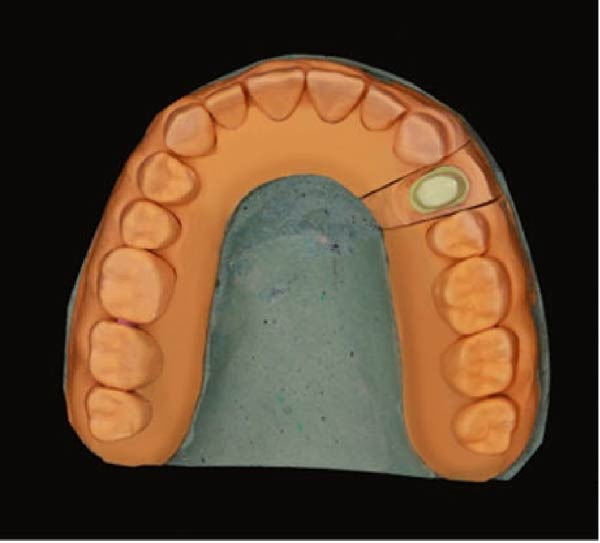
(A)

**Figure:**
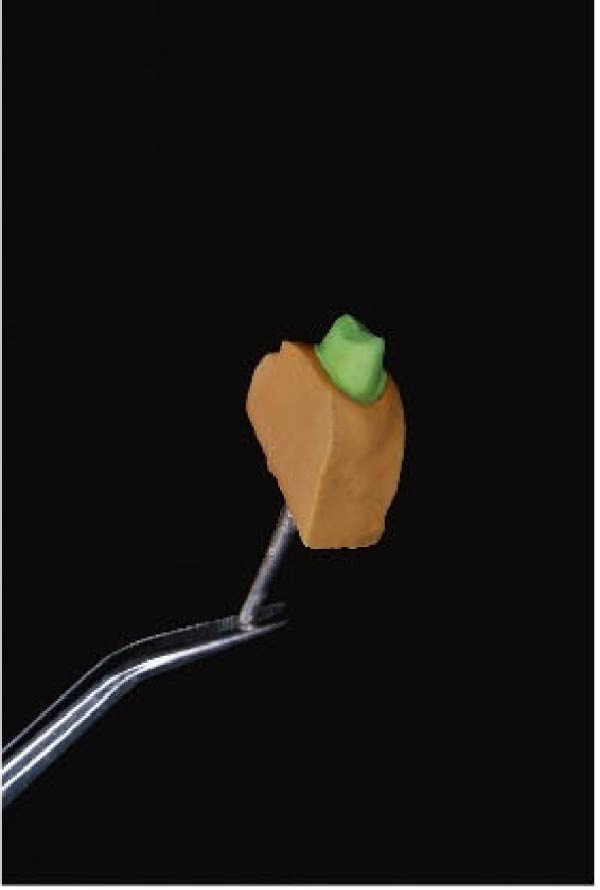
(B)

**Figure:**
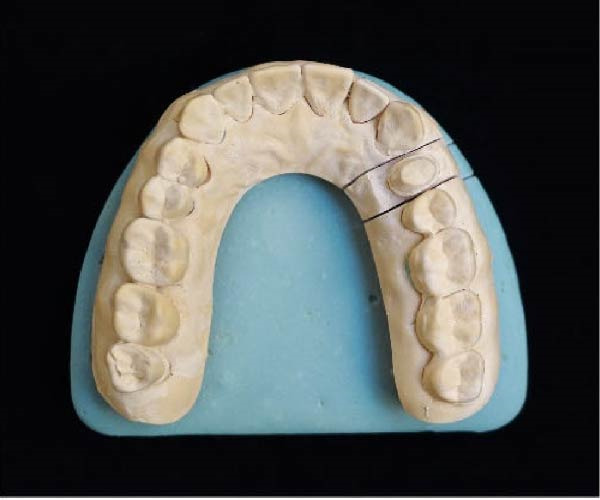
(C)

**Figure:**
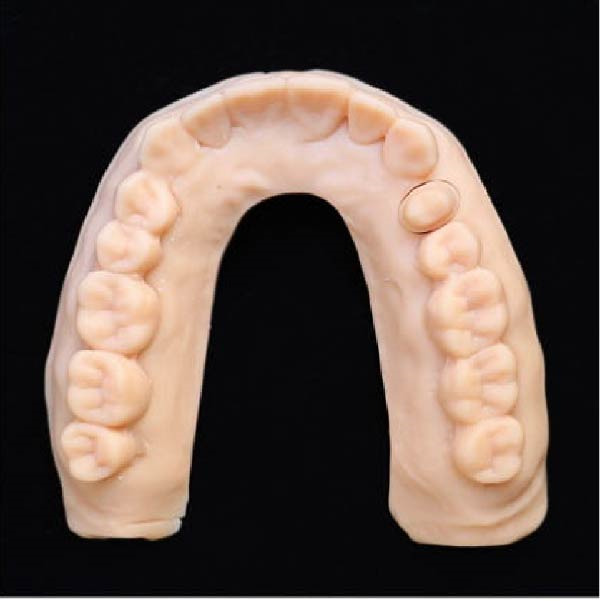
(D)

The ditching was conducted using a No. 2 round bur on a contra‐angle handpiece, and the prepared die is presented in Figure 2B.

### 2.3. Conventional Technique

Ten impressions were taken from the maxillary arch using a two‐step putty‐wash technique. A single‐colored Type IV die‐stone was employed to fill the impression. A brass pin was installed, and a separating agent was applied after approximately 30–45 min. The base stone was added to the cast and subsequently set and separated from the die using a dental lab plaster saw (Figure 2C). A no. 2 round bur attached to a contra‐angle handpiece was carefully employed to reveal the finish lines.

### 2.4. Digital Technique

Ten digital scans of the prepared typodont were taken using an Up600 intraoral scanner. The same operator performed the scanning procedures. The scanning procedure began from the occlusal surface of the second molar and extended to the contralateral side. The scans were saved as STL (stereolithography) files, used for creating digital models in the Exocad 2024 dental CAD program (Exocad GmbH), enabling modifications to improve fit and accuracy. The dies were separated from the digital casts using Exocad, and subsequently, 3D printed with a Renfert 3D printer (Renfert GmbH, Hilzingen, Germany). After printing, the dies were refined using isopropyl alcohol to eliminate the uncured resin, followed by final UV‐curing to improve the material’s qualities (Figure 2D).

### 2.5. Preparation of the Reference Model

The prepared teeth were separated from the typodont and scanned with a scanner (ATOS Core‐80; GOM GmbH) to create a reference (Figure 3A). The marginal accuracy was evaluated using ATOS software (Atos Professional; GOM, ZEISS company, Braunschweig, Germany).

Figure 3(A) Reference model displayed in ATOS professional 2018 software. (B) A digital scan taken by a laboratory scanner and exported as an STL file.

**Figure:**
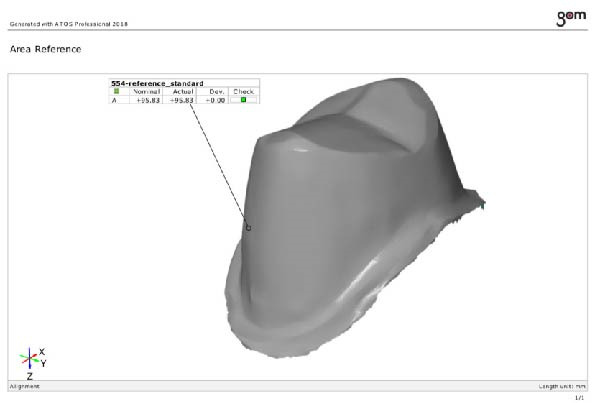
(A)

**Figure:**
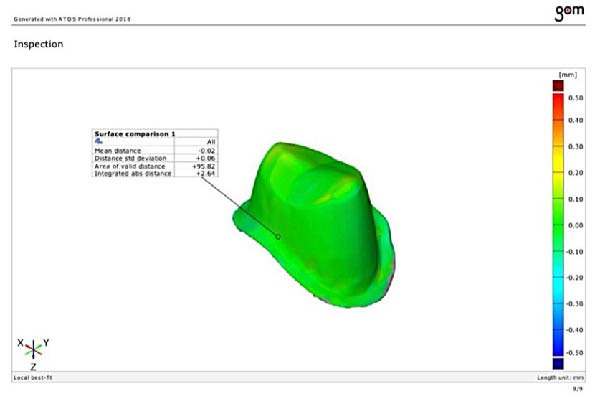
(B)

### 2.6. Assessment Procedure

Each die was scanned using a laboratory scanner (Figure 3B), and the finish lines were evaluated focused on the following parameters.

#### 2.6.1. Area of Valid Distance

It represents the portion of the finish line within an acceptable accuracy range. The software was programmed to compare the finish line area of each die to the reference model.

#### 2.6.2. Distance Standard Deviation

It measures the difference between the reference model and the finish line of the die.

#### 2.6.3. Integrated Absolute Distance

This parameter was calculated to measure the total inscribed volume of surface differences between each die and reference model.

#### 2.6.4. Mean Difference

The software calculated the mean deviation along the finish line by averaging the distance between the die and the finish line. This average could indicate the accuracy of each casting method.

The same trained operator conducted all analyses to ensure consistent measurements. To provide an impartial assessment, these parameters were meticulously measured at multiple points along the finish line using ATOS Professional 2018 software, demonstrating the thoroughness of our process and the reliability of our results.

All groups were evaluated, and pairwise comparisons were performed to identify significant differences between these techniques. Additionally, all groups were compared with the reference model regarding the area of valid distance. This comparison helped to determine whether each technique’s results fell within the acceptable accuracy range.

### 2.7. Statistical Analysis

The numerical data were assessed for normality distribution using the Shapiro–Wilk test. Due to the non‐normal distribution of our data, they were presented as medians and interquartile ranges. For the same reason, we used the Kruskal–Wallis test to compare the three groups’ medians. We employed the Wilcoxon rank‐sum (Mann–Whitney U) test for pairwise group comparisons after the significant Kruskal–Wallis test. A two‐sided *p*‐value of 0.05 or lower was considered statistically significant for the Kruskal–Wallis test and 0.017 for every Mann–Whitney U test due to adjustment for three pairwise comparisons.

## 3. Results

Tables 1 and 2 compare techniques across four parameters: area of valid distance, distance standard deviation, integrated absolute distance, and mean distance.

**Table 1:** Comparison of finish line quality metrics among digital, conventional, and DCDS techniques.

Variables	Group			*p*‐Value
	Digital	Conventional	DCDS	
N	10 (33.3%)	10 (33.3%)	10 (33.3%)	‐‐‐
Area of valid distance; median (IQR)	94.8 (94.7–94.9)	93.1 (90.9–95.2)	95.3 (95.2‐–95.8)	0.002^∗^
Distance standard deviation; median (IQR)	0.1 (0.1–0.1)	0.1 (0.1–0.1)	0.1 (0.1–0.1)	0.428
Integrated absolute distance; median (IQR)	3.1 (2.93.4)	2.3 (1.6–2.7)	2.2 (1.5–3.4)	0.007^∗^
Mean distance; median (IQR)	0.0 (0.0–0.0)	0.0 (0.0–0.0)	0.0 (‐0.0–0.0)	0.561

Abbreviation: DCDS, double‐colored die‐stone.

^∗^Indicates a statistically significant difference (*p* < 0.05).

**Table 2:** Pairwise comparison of *p*‐values for finish line accuracy across methods.

Variables	*p*‐Value		
	Digital and conventional	Digital and DCDS	Conventional and DCDS
Area of valid distance; median (IQR)	0.382	<0.001 ^∗^	0.005 ^∗^
Integrated absolute distance; median (IQR)	<0.001 ^∗^	0.086	0.839

Abbreviation: DCDS, double‐colored die‐stone.

^∗^ *p*‐Value <0.05, considered statistically significant.

Area of valid distance: According to Table 1, the highest value for the area of valid distance was observed with the DCDS technique (95.3). A significant difference was found between the groups (*p* = 0.002), with pairwise comparisons revealing substantial differences between the digital and DCDS groups (*p*  < 0.001) and conventional and DCDS groups (*p* = 0.005). No significant difference was found between digital and DCDS technique.

Distance standard deviation: No significant differences between the groups were observed in this parameter (*p* = 0.428).

Integrated absolute distance: The highest value for integrated absolute distance was observed in the digital group (3.1). There was a significant difference between the groups in this parameter (*p* = 0.007). Table 2 shows pairwise comparisons, which showed a significant difference between the digital and conventional techniques (*p*  < 0.001).

Mean distance: No significant difference was found in this parameter between the groups (*p* = 0.561).

Table 3 compares the three techniques with the reference model. These results suggest that all three methods substantially deviated from the reference model, with a median of 95.83 for the area of valid distance. Significant differences were observed in the area of valid distance between the digital, conventional, and DCDS techniques (*p* = 0.002, *p* = 0.002, and *p* = 0.011, respectively). The DCDS technique showed the closest value to the reference model (95.3).

**Table 3:** Comparison of finish line accuracy between digital, conventional, and DCDS methods against the reference model.

Variables	*p*‐Value		
	Digital	Conventional	DCDS
Area of valid distance	0.002^∗^	0.002^∗^	0.011^∗^
Wilcoxon signed‐rank test for testing the median of each group equals 95.83			

Abbreviation: DCDS, double‐colored die‐stone.

^∗^Indicates a statistically significant difference (*p* < 0.05).

## 4. Discussion

This study was the first to introduce the DCDS technique to facilitate the identification of the finish line in dental casts. We evaluated the casts according to these criteria: the area of valid distance, distance standard deviation, integrated absolute distance, and mean distance. Our results showed that the area of valid distance was significantly higher in the DCDS technique than in the digital technique. The digital technique demonstrated a substantially higher value of integrated absolute distance than the conventional technique. Furthermore, we revealed a significant deviation from the reference model for all three techniques, with the DCDS technique exhibiting the closest value to the reference model.

Capturing marginal detail is crucial in the impression‐taking process. Inaccurate recording of the finish line could lead to insufficient prosthesis fit [14]. In recent years, digital fabrication techniques, such as milling and 3D printing, have become widely popular and have effectively replaced the conventional method. 3D printing is emerging as an important growth field within digital technology [15]. Despite the high precision levels offered by 3D printing, significant investment in specialized tools and training is often required [11]. In contrast, the DCDS technique offers a cost‐effective alternative, creating high‐quality outcomes that enhance the finish line visibility and margin detection, especially in labs with limited access to digital equipment.

Previous studies compared digital and conventional methods [5, 6, 8]. For instance, Júnio et al. [5] demonstrated that frameworks created with digital impressions exhibited better internal fit than conventional frameworks. Likewise, Svanborg et al. [8] stated that the digital impression technique is more accurate and produces 3‐unit fixed dental prostheses with a significantly better fit than the vinyl polysiloxane technique. Similarly, Aly and Mohsen [6] reported that the accuracy of SLA‐printed casts generated from 3Shape intraoral scanners showed minimal errors compared to conventional stone casts. Our results align with these findings and show a significantly higher integrated absolute distance in the digital method than in the conventional method.

While intraoral scanning offers several benefits, its application is limited by specific challenges. For example, it can be difficult to achieve a clear scan when blood or saliva is present in the mouth. Furthermore, some intraoral scanners are unsuitable for precisely determining the extent of mobile tissues or capturing soft‐tissue morphology [16]. Our research introduced a technique that outperforms the digital approach in capturing the area of valid distance. Our findings showed that using DCDS to fill the impressions for fixed prostheses reduces the errors in finish‐line detection.

However, this is the first study to apply DCDS to prepare dental casts. Dual‐color systems are extensively used in medical applications to increase the accuracy and visibility of crucial components. For example, dual‐color fluorescence imaging facilitates the visualization of angiogenesis and tumor development [17]. Furthermore, the dual color bioreporter technique is a rapid and efficient prescreening technology to distinguish both androgenic and antiandrogenic substances [18]. These examples utilize multiple colors to maximize precision and decrease errors, similar to how DCDS improves the clarity of the finish line in dental casts.

The current study presents an alternative approach to fabricating prosthetic restorations, which offers several advantages. First, the DCDS technique enhances the visibility of the finish line during the ditching process. Additionally, technicians could precisely align the die’s finish line with the mold’s original margin. This technique also has the potential to improve the marginal fit of the frames due to reduced chairside adjustments.

This approach offers technical advantages and stands out for its financial feasibility. It provides a cost‐effective alternative to resin casts, especially for laboratories lacking advanced digital equipment, allowing them to achieve high‐quality results with minimal resources.

While demanding additional training and resources, this technique offers a potential method to significantly enhance the quality of prosthetic restorations in complicated cases.

Despite the small sample size, this pilot study revealed that the DCDS technique is an appropriate alternative to the conventional technique when digital equipment is unavailable. This approach could also lead to better outcomes than digital methods regarding the area of valid distance. Furthermore, as a pilot study, this research has limitations caused by the small sample size, which restricted the generalizability of our conclusion to broader applications. Future studies with larger sample sizes are essential to validate the efficacy of the DCDS technique. Future studies should also evaluate the effect of the DCDS technique on patient outcomes, cost‐effectiveness, and long‐term durability.

## 5. Conclusion

This study’s findings suggest that the DCDS method could be a superior alternative to conventional techniques to facilitate the ditching process and detect the finish line on die casts. Although the DCDS technique is comparable to digital methods, it could improve the accuracy of the finish line in the final die by minimizing the risk of errors during finish line detection on the cast. This improvement could result in greater satisfaction for dentists and patients.

## Ethics Statement

Ethical approval was not required for this in vitro study, as no human subjects, patient data, or animal models were used.

## Conflicts of Interest

The authors declare no conflicts of interest.

## Author Contributions

**Ahmad Toumaj**: conceptualization, project administration, investigation, visualization, writing – review and editing, methodology. **Akram Ghannadpour**: investigation, supervision. **Mahtab Mottaghi**: investigation, writing – original draft, software. **Mirkamal Hosseiny**: investigation, writing – review and editing. **Seyed Mehdi Ziaei**: investigation, writing – review and editing. **Mona Saligherad**: investigation. **Kiarash Kiani**: investigation.

## Funding

The authors received no financial support for the research, authorship, and/or publication of this paper.

## Data Availability

The data that support the findings of this study are available from the corresponding author upon reasonable request.
